# The Early Endocrine Stress Response in Experimental Subarachnoid Hemorrhage

**DOI:** 10.1371/journal.pone.0151457

**Published:** 2016-03-23

**Authors:** Christoffer Nyberg, Torbjörn Karlsson, Lars Hillered, Mats Stridsberg, Elisabeth Ronne Engström

**Affiliations:** 1 Department of Neuroscience, Section of Neurosurgery, Uppsala University, Uppsala, Sweden; 2 Department of Surgical Sciences, Section of Anesthesiology and Intensive care, Uppsala University, Uppsala, Sweden; 3 Department of Medical Sciences, Uppsala University, Uppsala, Sweden; Glasgow University, UNITED KINGDOM

## Abstract

**Introduction:**

In patients with severe illness, such as aneurysmal subarachnoid hemorrhage (SAH), a physiologic stress response is triggered. This includes activation of the hypothalamic-pituitary-adrenal (HPA) axis and the sympathetic nervous system. The aim of this study was to investigate the very early responses of these systems.

**Methods:**

A porcine animal model of aneurysmal SAH was used. In this model, blood is injected slowly to the basal cisterns above the anterior skull base until the cerebral perfusion pressure is 0 mm Hg. Sampling was done from blood and urine at -10, +15, +75 and +135 minutes from time of induction of SAH. Analyses of adrenocorticotropic hormone (ACTH), cortisol, aldosterone, catecholamines and chromogranin-A were performed.

**Results:**

Plasma ACTH, serum cortisol and plasma aldosterone increased in the samples following induction of SAH, and started to decline after 75 minutes. Urine cortisol also increased after SAH. Urine catecholamines and their metabolites were found to increase after SAH. Many samples were however below detection level, not allowing for statistical analysis. Plasma chromogranin-A peaked at 15 minutes after SAH, and thereafter decreased.

**Conclusions:**

The endocrine stress response after aneurysmal SAH was found to start within 15 minutes in the HPA axis with early peak values of ACTH, cortisol and aldosterone. The fact that the concentrations of the HPA axis hormones decreased 135 minutes after SAH may suggest that a similar pattern exists in SAH patients, thus making it difficult to catch these early peak values. There were also indications of early activation of the sympathetic nervous system, but the small number of valid samples made interpretation difficult.

## Introduction

In aneurysmal subarachnoid hemorrhage (SAH), blood is leaking from the arterial cerebral blood circulation into the subarachnoid space leading to increased intracranial pressure (ICP) and subsequent reduction of the cerebral perfusion pressure (CPP). The blood is commonly distributed in the basal cisterns with close relation to the pituitary gland. Disturbances in the pituitary function have been demonstrated, both in the acute phase [1–5] and in long-term follow-up [6–9]. This could be a consequence of a general cerebral injury caused by the sudden increase in ICP, decrease in CPP and transient global brain ischemia at the time of aneurysm rupture. It is also possible that the proximity of the blood to the pituitary gland and the hypothalamus directly affects the pituitary function.

In aneurysmal SAH, there is a sudden, severe ischemic impact on the brain. This event triggers an immediate response in order to restore the cerebral blood circulation. The response includes activation of the sympathetic nervous system [10–12] and of the hypothalamic-pituitary-adrenal (HPA) axis.

Activation of the HPA axis is seen with critical illness in general and is considered to be a physiologic response to stress [13], leading to elevated blood cortisol levels and most likely of great importance to meet the challenges of critical illness, such as SAH.

Elevated cortisol concentrations have been demonstrated early in the acute phase in SAH patients, but it remains unclear whether the increase of cortisol is impaired because of pituitary dysfunction or is adequate in response to the trauma. Low levels of cortisol have also been demonstrated, both in the acute phase and in long-term follow-up. It has also been found that the normal diurnal cortisol excretion may be altered in patients with SAH [5, 14].

In several studies, indications of early pituitary deficiency have been found [2, 3] and cortisol concentrations have been shown not to be directly linked to severity of the disease in SAH [6], also indicating a possible insufficiency in the pituitary-adrenal response. It has also been demonstrated that episodes of low cortisol are associated with increased risk of poor outcome [2].

In patients, blood cortisol levels can be analyzed when the patient presents at the hospital and has been diagnosed with subarachnoid hemorrhage, typically more than one hour after the actual time of aneurysm rupture. The dynamics of cortisol as well as other stress-related hormones in the very early phase of SAH is not known. These very early mechanisms after onset of SAH are difficult to study in humans because patients are generally admitted to a local hospital, diagnosed with SAH and thereafter transferred to a secondary hospital with a considerable time delay.

The aim of this study was to investigate the endocrine stress response immediately after onset of the disease. Measurements of blood concentrations of adrenocorticotropic hormone (ACTH) and cortisol are included, as well as aldosterone. In order to examine the sympathetic stress response, catecholamines and their metabolites were examined in urine. This was complemented with measurements of chromogranin-A in plasma, a substance co-released with catecholamines and a marker of sympathetic activity [15, 16].

We used a porcine animal model of SAH that was developed to mimic conditions of aneurysm rupture to investigate the nature of the endocrine stress response in the ultra-early phase after SAH [17]. In this model, autologous blood is injected close to the anterior skull base, a location commonly affected by aneurysmal SAH. It may also be important to use blood rather than artificial cerebrospinal fluid. Blood is composed by a wide variety of substances and is believed to play a major role in the development of vasospasm in the course of SAH [18]. It has been demonstrated that substances in blood activates neurons when injected into the subarachnoid space [19, 20].

## Material and Methods

### Animals, anesthesia and SAH induction

Experimental SAH was induced in pigs, a detailed description of this procedure has been previously published [17]. 11 pigs under general anesthesia underwent experimental SAH by autologous blood injection to the anterior skull base. Intracranial pressure and cerebral perfusion pressure were monitored during the injection; in order to mimic the conditions of aneurysm rupture in patients, CPP was kept around 0 mm Hg for one minute by slowly continuing the injection.

The animals were of mixed breed and of both sexes. The age of the pigs was 8–10 weeks with a mean weight of 24.9 (23.1–28.8) kg. At induction of anesthesia, the animals received a bolus fluid infusion with 30 mL/kg of Ringer-acetat (Fresenius Kabi, Uppsala, Sweden). Thereafter, fluid infusion was administered with Ringer-acetat at 8 mL/kg/h and Rehydrex 25 mg/mL (Fresenius Kabi) at 10 mL/kg/h. Before transportation to the laboratory, the animals were given intramuscular injections of 50 mg xylazine (Rompun vet, Bayer Health Care, Leverkusen, Germany).

Induction of anesthesia was performed with intramuscular injections of tiletamine 3 mg/kg and zolazepam 3 mg/kg (Zoletil 100, Virbac, Carros, France), xylazine 2.2 mg/kg (Rompun vet, Bayer Health Care) and atropine 0.04 mg/kg (Atropin Mylan, Mylan, Stockholm, Sweden) in combination with intravenous injections of ketamine 100 mg (Ketaminol vet, Intervet International, Boxmeer, Netherlands) and morphine 1 mg/kg (Morfin Meda, Meda, Solna, Sweden). Maintenance of anesthesia was achieved by continuous intravenous infusion of ketamine 20 mg/kg/h, morphine 0.5 mg/kg/h and rocuronium bromide 2 mg/kg/h (Esmeron, NV Organon, Oss, Netherlands).

Immediately after the experiment was finished, the animals were euthanized with potassium chloride while still under anesthesia.

### Blood and urine sampling

Samples of blood and urine were collected from each animal at -10, +15, +75 and +135 minutes from time of SAH induction. The blood samples were drawn from an arterial line. Ten minutes before each sampling, urine collection started and samples were taken for analysis from this volume.

The blood samples were analyzed for concentrations of serum cortisol, plasma ACTH, plasma aldosterone and plasma chromogranin-A. Urine samples were analyzed for concentrations of cortisol, creatinine, adrenalin, noradrenalin and metabolites from catecholamines.

### Biochemical methods

Measurements of cortisol were performed on automatic immune analyser (Cobas e601, Roche Diagnostics, Basel, Switzerland). The total assay variation was less than 9%. Measurements of ACTH were performed on automatic immune analyser Immulite 2000 XPi (Siemens, Los Angeles, CA, USA). The total assay variation was less than 6%. Measurements of creatinine were performed on automatic immune analyser Architect Ci8200^®^ analyser (Abbott, Abbot Park, IL, USA). The total assay variation was less than 4%. Measurements of aldosterone were performed with a manual radioimmunoassay (Coat-a-Count-Bio International, Codolet, France). The total assay variation was less than 9%. Measurements of catecholamines were performed on a high-performance liquid chromatography system [21]. The total assay variation was less than 10%. Measurements of chromogranin-A were performed with an in-house radioimmunoassay [22]. The assay, based on human amino acid sequences, measure the N-terminal part of the molecule, which has 100% cross reactivity to porcine chromogranin-A. The total assay variation was less than 10%. All analyses were performed at the routine laboratory of the Department of Clinical Chemistry at the University Hospital in Uppsala. The laboratory is certified by a Swedish government authority (Swedac).

### Statistics

Statistical analyses were performed with the software STATISTICA 10 (StatSoft, Inc. Tulsa, OK). The change of concentrations of hormones from baseline values were evaluated with the Wilcoxon matched pairs test. The Friedman test was used for analysis of variance of hormone concentrations over time. Values of p<0.05 were considered significant.

### Ethics

The study protocol was approved by the Uppsala Institutional Review Board for Animal Experimentation (Permit number C187/11). The pigs were handled according to the guidelines of the Swedish National Board for Laboratory Animals and the European Convention of Animal Care.

## Results

As described in the previously published material [17], there were two different patterns in the animals after SAH induction. In nine animals, ICP and CPP were restored towards pre-injury levels shortly after SAH induction followed by a gradual slow increase in ICP during the rest of the monitoring time. This suggests a transient ischemia followed by a disturbed circulation of cerebrospinal fluid. In the other two animals, ICP remained elevated and CPP remained negative, compatible with persisting ischemia. These findings were also supported by cerebral microdialysis results.

There was also a clear distinction between the two groups described above in regard to concentrations of hormones connected to endocrine stress response; the two pigs with persisting ischemia were therefore analyzed separately from the other nine pigs.

The concentrations of analyzed hormones are presented in Table 1.

**Table 1:** Hormone concentration in the animals with transient ischemia (n = 9) and significance testing of change from baseline (-10 minutes) using Wilcoxon matched pairs test.

	Minutes after SAH	Mean	Median	Range	SD	*p*
Plasma ACTH	-10	18.8	7	5.7–101	31	
*ng/L*	+15	37.5	24	9.1–101	30	0.012
	+75	72.2	71	7.5–126	38	0.051
	+135	64.8	58	10.0–117	35	0.028
Serum Cortisol	-10	36.1	28	14.3–89.0	23	
*nmol/L*	+15	156.8	181	13.9–252.0	80	0.011
	+75	240.6	275	5.82–329.0	98	0.011
	+135	202.2	224	6.57–327.0	101	0.017
Plasma Aldosterone	-10	112.7	101	56–248	60	
*pmol/L*	+15	196.9	200	84–311	83	0.012
	+75	268.9	294	101–409	94	0.012
	+135	221.3	221	101–426	109	0.028
Plasma Chromogranin-A	-10	0.140	0.117	0.103–0.219	0.045	
*nmol/L*	+15	0.164	0.151	0.103–0.301	0.059	0.441
	+75	0.111	0.107	0.037–0.208	0.055	0.139
	+135	0.102	0.102	0.029–0.196	0.055	0.069
Urine Cortisol	-10	131.8	142	35–232	79	
*nmol/L*	+15	95.1	74	26–204	64	0.051
	+75	255.3	234	43–436	130	0.066
	+135	379.4	415	16–710	212	0.036
Urine Cortisol/cretatinine	-10	40.0	36	16.9–69	16	
*μmol/mol*	+15	38.6	32	15.3–68	18	0.678
	+75	97.1	88	9.9–203	66	0.021
	+135	167.6	163	15.9–300	96	0.017

### ACTH

**Fig 1:**
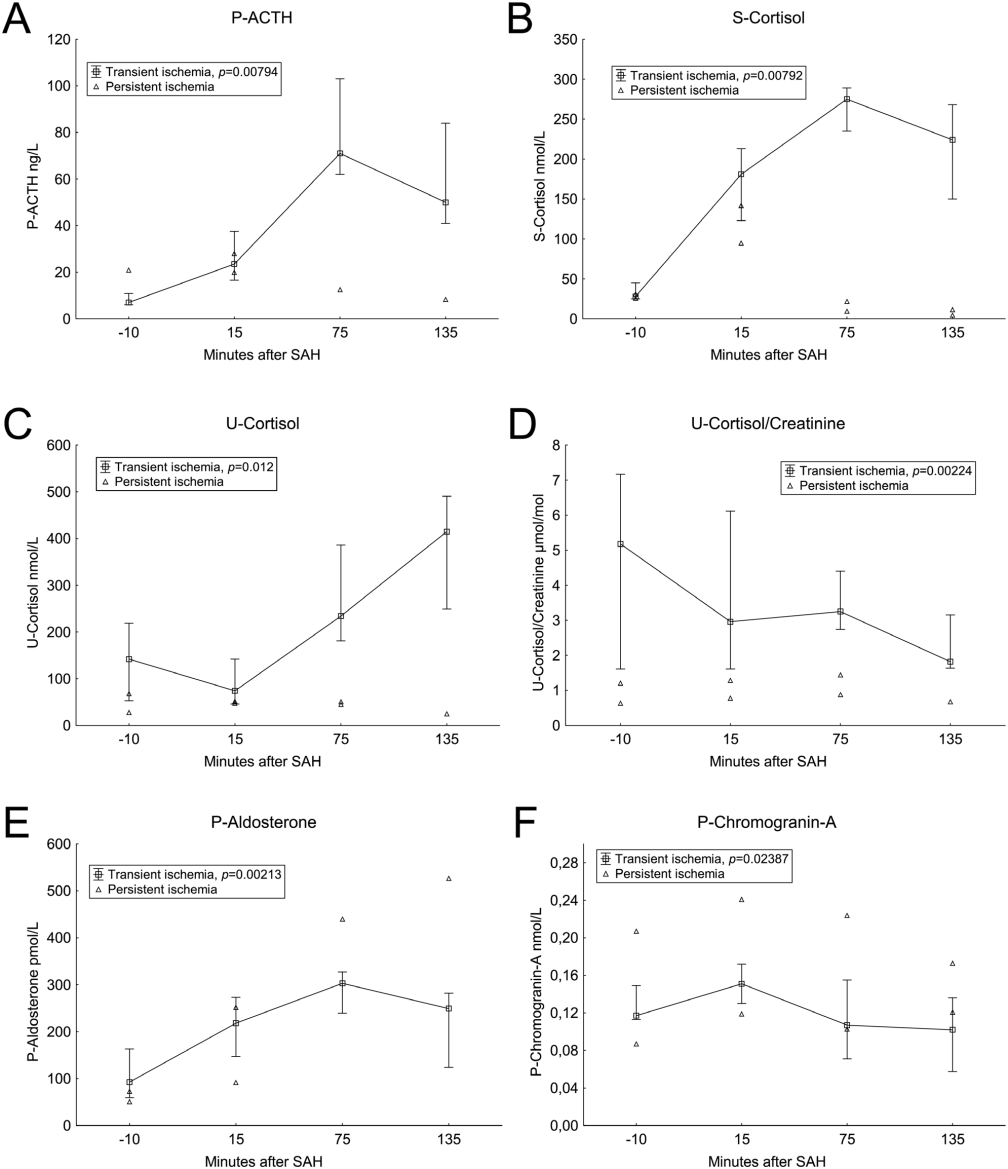
Blood hormones. Median values with 25^th^ and 75^th^ percentiles. Animals with persistent ischemia are displayed separately. Significance testing of change over time with the Friedman test. Time scale is non-linear.

After induction of SAH, plasma ACTH levels increased in the two following measurements. The highest concentrations of ACTH were found in the sample taken 75 minutes after SAH. In the last sample (135 minutes after SAH) the levels of ACTH had started to decrease, but the levels were still elevated compare to baseline values. The two animals with persisting ischemia did not show an elevation of ACTH levels after SAH. The dynamics of ACTH is shown in Fig 1A.

### Cortisol

Cortisol concentrations in serum increased in the same pattern as ACTH with increasing levels after SAH, peaking at 75 minutes after SAH induction, see Fig 1B. The two animals with persisting signs of ischemia had an increase of cortisol levels in the measurements 15 minutes after SAH, but this was followed by decreasing levels. In urine, cortisol levels increased throughout the experiment (Fig 1C). However, when the quotient with urine creatinine was analyzed, the urine cortisol levels after SAH did not increase compared with baseline values (Fig 1D).

### Aldosterone

Concentrations of plasma aldosterone increased after SAH in a pattern similar to ACTH and cortisol, with the highest concentrations after 75 minutes. The two animals with persisting ischemia did not differ distinctively from the rest of the animals. Aldosterone concentrations are shown in Fig 1E.

### Catecholamines and Chromogranin-A

**Fig 2:**
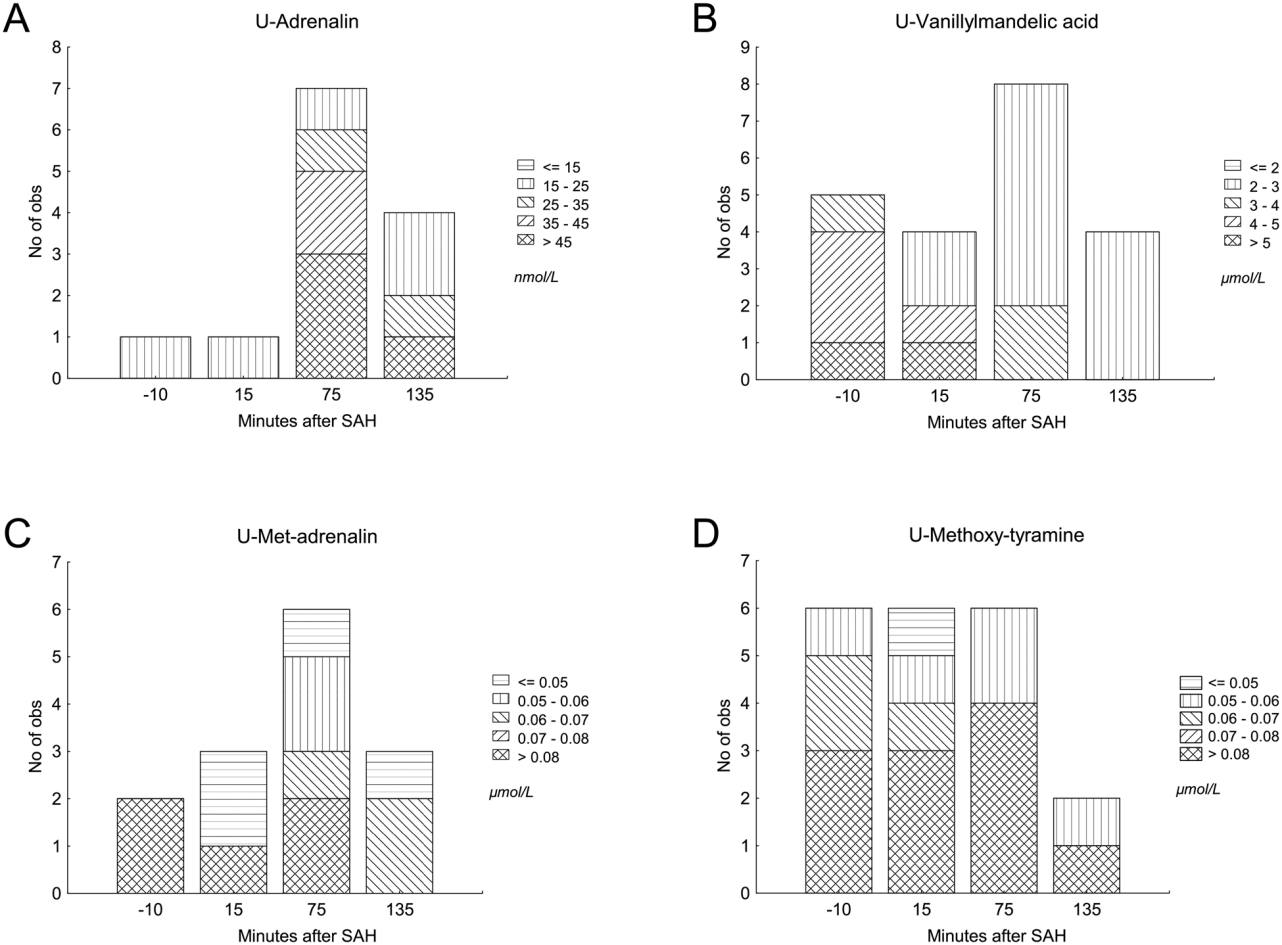
Catecholamines in urine. Catecholamines and metabolites in urine in animals with transient ischemia. Samples with detectable concentrations are shown.

Adrenalin, noradrenalin and their metabolites were found in some urine samples. However, many samples were below detection level. Adrenalin, met-adrenalin, methoxy-tyramine and vanillylmandelic acid were the only substances found in more than sporadic samples. Noradrenalin and its specific metabolites were only detected in a very small number of samples. The concentrations in urine of adrenalin and its metabolites are shown in Fig 2. No statistical analysis was performed because of the small number of samples.

Chromogranin-A concentrations in plasma (Fig 1F) increased after SAH induction, and thereafter decreased in the two following measurements.

## Discussion

### Hypothalamic-pituitary-adrenal axis response

Elevated levels of both ACTH and cortisol have been demonstrated in patients early in the course of SAH [1, 5, 6]. In the present study, the rise of ACTH and cortisol seem to be very rapid after onset of disease. Both ACTH and cortisol shows elevated concentrations in the first measurement 15 minutes after SAH induction, and peaks at the measurement 75 minutes after SAH. In the last measurement, 135 minutes after SAH, ACTH and cortisol decreased compared to the peak vale. This indicates that the initial peak of these two hormones may be very early. The dynamics of aldosterone was very similar to ACTH and cortisol, which implicates that this rapid and early rise of aldosterone was most likely stimulated by ACTH, rather than by other mechanisms.

It has previously been well established that activation of the HPA axis occurs in patients with SAH. However, these measurements were not done in the ultra-early phase after onset of disease. Our results are in analogy with these results, showing a very early and rapid response after SAH. It is interesting that concentrations of all analyzed hormones in blood of the HPA axis decreased in the last measurement. This fact raises a suspicion that the peak of these stress hormones could be very early and difficult to catch in patients because of the delay from onset of disease to blood sampling.

The measurements of cortisol in urine also show increasing concentrations, but the quotient of cortisol and creatinine is not increasing. These results make the interpretation of cortisol in urine difficult, and could be a result of a methodological problem as described below.

### Sympathetic stress response

Many samples of catecholamines and their metabolites in urine were below detection level, not allowing for statistical analysis. However, adrenalin, met-adrenalin, methoxy-tyramine and vanillylmandelic acid (VMA) were found in some cases, showing an increasing trend at 75 minutes after SAH. Met-adrenalin and met-noradrenalin are degradation products of adrenalin and noradrenalin, respectively. These two metabolites are further degraded to VMA and excreted in urine. Methoxy-tyramine is a degradation product of another catecholamine, dopamine. The findings of catecholamines and their metabolites support that there was an early sympathetic stress response following the induction of SAH.

Because of short half-life and other difficulties in measurements of catecholamines, chromogranin-A is sometimes used as a marker of sympathetic activity. Chromogranin-A is co-released with adrenalin and noradrenalin, and also with other neuroendocrine hormones.

In this study, plasma chromogranin-A showed an increasing trend and thereafter decreased. The measurements at 75 and 135 minutes after SAH were lower than baseline values at 10 minutes before SAH. This finding may be connected with the urinary metabolites of catecholamines that also were higher at baseline, possibly because of too short time after surgical preparation.

### Endocrine stress response after SAH

In this study, we found evidence of an endocrine stress response after experimental SAH, involving both the HPA axis and the sympathetic nervous system. There is evidence in the literature that the stress response in patients with SAH involves the endocrine systems described here, but also other components, including brain natriuretic peptide (BNP) [1]. The stress response in patients is most likely essential to survive the challenges of life-threatening disease, but the complicated mechanisms in the stress response may also be involved in many complications of SAH, such as hypovolemia, cerebral vasospasm and delayed ischemic neurological deficits (DIND). The results in our study add knowledge to some aspects of the endocrine stress response immediately after onset of SAH. However, further studies are needed to fully describe the stress response and be able to understand which parts of the stress response that are inadequate and increase the risk of poor outcome.

### Limitations of the study

Surprisingly, the concentrations of some metabolites of catecholamines were higher in baseline measurement 10 minutes before SAH than 15 minutes after SAH. This is possibly an effect of the induction of anesthesia and the surgical preparation before start of the experiment. After the surgical preparation, there was a period of 30 minutes before starting the intervention. To avoid influences on concentrations of catecholamines and their metabolites, it might be beneficial to extend this period of resting state in future experiments.

The analysis of catecholamines and cortisol in urine was done by collecting a small volume of urine at the time of sampling. It may have been better to collect urine continuously between the sampling times and draw a sample from that volume. We also noticed that urine production differed substantially between the animals, although the volumes were not precisely measured. Some animals did not produce urine at all in the end of the experiment. These two factors most likely also influenced the measurements of catecholamines. Creatinine in urine was also analyzed, showing large differences between different animals and measurements. This also supports the idea that urine samples between and within animals are not easily compared.

The peak of hormones in the HPA axis was very early and the values had started to decrease in the last measurement. It would have been interesting to continue the sampling a longer time period after SAH, covering the entire time when patients are not possible to study to find out whether the decrease of hormones was temporary or not.

## Conclusions

The endocrine stress response in the studied porcine model of aneurysmal SAH was found to start within the first 15 minutes after SAH induction. The response includes activation of the HPA axis with peaking blood values of ACTH, cortisol and aldosterone early after onset of disease. These very early responses have not been described previously and are very difficult to study in humans. Indications of a catecholamine response was also found, although the small number of cases and methodological issues made interpretation difficult.
